# Boosting Reversibility of Mn‐Based Tunnel‐Structured Cathode Materials for Sodium‐Ion Batteries by Magnesium Substitution

**DOI:** 10.1002/advs.202004448

**Published:** 2021-02-18

**Authors:** Xun‐Lu Li, Jian Bao, Yi‐Fan Li, Dong Chen, Cui Ma, Qi‐Qi Qiu, Xin‐Yang Yue, Qin‐Chao Wang, Yong‐Ning Zhou

**Affiliations:** ^1^ Department of Materials Science Fudan University Shanghai 200433 P. R. China

**Keywords:** cathode materials, Mg substitution, phase transitions, sodium‐ion batteries

## Abstract

Electrochemical irreversibility and sluggish mobility of Na^+^ in the cathode materials result in poor cycle stability and rate capability for sodium‐ion batteries. Herein, a new strategy of introducing Mg ions into the hinging sites of Mn‐based tunnel‐structured cathode material is designed. Highly reversible electrochemical reaction and phase transition in this cathode are realized. The resulted Na_0.44_Mn_0.95_Mg_0.05_O_2_ with Mg^2+^ in the hinging Mn‐O_5_ square pyramidal exhibits promising cycle stability and rate capability. At a current density of 2 C, 67% of the initial discharge capacity is retained after 800 cycles (70% at 20 C), much improved than the undoped Na_0.44_MnO_2_. The improvement is attribute to the enhanced Na^+^ diffusion kinetics and the lowered desodiation energy after Mg doping. Highly reversible charge compensation and structure evolution are proved by synchrotron‐based X‐ray techniques. Differential charge density and atom population analysis of the average electron number of Mn indicate that Na_0.44_Mn_0.95_Mg_0.05_O_2_ is more electron‐abundant in Mn 3d orbits near the Fermi level than that in Na_0.44_MnO_2_, leading to higher redox participation of Mn ions.

## Graphical abstract

Na_0.44_Mn_0.95_Mg_0.05_O_2_ as a novel cathode material for sodium‐ion batteries is designed and synthesized aiming to effectively facilitate Na^+^ diffusion and lower desodiation energy. Structure evolution of tunnel‐type cathode during high rate charging/discharging is revealed for the first time, enabling high rate capability and cycle stability with the help of Mg.

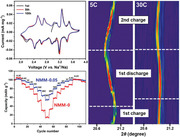

## 1 Introduction

Rechargeable sodium‐ion batteries (SIBs) are considered as the most appealing alternative to lithium‐ion batteries (LIBs) regarding the limited natural abundance and increasing consumption of Li resources. Similar to LIBs, Na^+^ can be the charge carrier to achieve electrochemical energy storage in SIBs.^[^ ^1^, ^2^, ^3^, ^4^, ^5^ ^]^ However, sluggish mobility of Na ions result in relative lower energy density and poorer kinetics than LIBs. Therefore, developing high‐performance cathode materials is essential for pushing the practical application of SIBs.

Over the past years, several kinds of cathode materials have been developed for SIBs, including the layer‐structured metal oxides, tunnel‐structured metal oxides, polyanion compounds, and organic materials.^[^ ^6^, ^7^, ^8^, ^9^ ^]^ Among them, tunnel‐structured Na_0.44_MnO_2_ cathode is a promising cathode material, first developed by Hagenmuller's group.^[^ ^10^ ^]^ Na ions in Na_0.44_MnO_2_ can shuttle through the large “S shape” tunnels reversibly. However, it suffers from structural degradation during longtime Na^+^ insertion and extraction. To solve the existing problems, plenty of works have been done in the recent years. Wang et al. designed Ti‐substituted Na_0.44_[Mn_0.66_Ti_0.34_]O_2_ and Na_0.66_[Mn_0.66_Ti_0.34_]O_2_ cathodes for aqueous sodium‐ion batteries. They revealed that Ti substitution could tune the charge ordering property and reaction pathway, significantly smooth the charge/discharge curves and improve cycle stability.^[^ ^11^, ^12^ ^]^ Xu et al. reported that Fe substitution in the tunnel Na_0.61_[Mn_0.61−_ *_x_*Fe*_x_*Ti_0.39_]O_2_ could realize Fe^3+^/Fe^4+^ redox couple in tunnel‐structured cathodes, achieving a high working voltage of 3.56 V with a usable capacity of ≈90 mAh g^–1^.^[^ ^13^ ^]^ Latter, Wang et al. successfully introduced F^–^ to enlarge the sodium diffusion paths of Na_0.66_[Mn_0.66_Ti_0.34_]O_2_, effectively inhibiting structural degradation (85 mAh g^−1^ is maintained at 2 C after 1000 cycles) and promoting electrochemical kinetics,^[^ ^14^ ^]^ especially enhancing the low temperature performance at −20 °C. Even so, there are still some unstable structure transformation for Mn‐based cathode materials due to the unavoidable Jahn–Teller distortion, which limits their further applications.^[^ ^15^, ^16^, ^17^ ^]^

As a kind of environmental‐friendly alkaline earth metal, Mg^2+^ has similar ionic radius as Mn^3+^ but are more stable in electronic structure. Mg^2+^ has been widely used for doping or substituting in cathode materials to improve their performance in electrochemical sodium storage.^[^ ^18^, ^19^ ^]^ In P2‐Na_0.67_Mn_1−_ *_y_*Mg*_y_*O_2_ (*y* = 0, 0.05, 0.1),^[^ ^20^ ^]^ increasing the amount of Mg^2+^ could smooth the electrochemical curve and mitigate Jahn–Teller effect. Due to the similar electronic structure but smaller ionic radius properties than Na^+^, Mg^2+^ can substitute into both Na sites and transition metal sites in P2‐Na_0.7_Mg_0.05_[Mn_0.6_Ni_0.2_Mg_0.15_]O_2_ compound. Mg ions in the prismatic sodium site play a role like “pillar” to stabilize the layered structure along *c*‐axis during Na^+^ intercalation/extraction. Although the electrochemical inert Mg^2+^ makes no contribution to the charge compensation for redox reaction, the formation of “Na‐O‐Mg” configuration can ionize the Mg—O bond, placing O 2*p* states relatively high in energy and inducing the oxygen redox without oxygen release.^[^ ^21^, ^22^ ^]^

Herein, we introduced Mg^2+^ into the hinging site allocated between small tunnel and “S shape” tunnel in tunnel‐structured Na_0.44_MnO_2_ to tune the structure evolution during sodiation/desodiation process and successfully relieve the lattice strain. The strategy of Mg doped NMM‐0.05 largely enhanced the kinetics of sodium storage, leading to superior long‐cycle stability and rate capability. Na_0.44_Mn_0.95_Mg_0.05_O_2_ keeps 67% capacity retention rate after 800 cycles at 2 C. Even at 20 C, a capacity of 60 mAh g^–1^ can still be obtained. X‐ray diffraction (XRD) and X‐ray absorption spectroscopy (XAS) demonstrated the high reversibility of structure evolution and charge compensation during charge and discharge. The immediate structure evolution during high‐rate cycling was also revealed by using synchrotron‐based time‐resolved XRD technique. Density functional theory (DFT) calculations illustrated the desodiation energy is decreased and the average charge density in Mn 3*d* orbits near the Fermi level is increased. Mg^2+^ doping could be an effective strategy for optimizing tunnel‐type cathode materials for SIBs.

## 2 Results and Discussion

**Figure 1:**
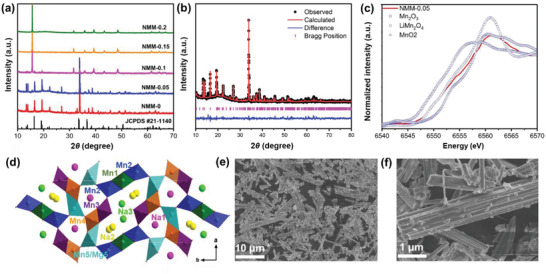
a) XRD patterns of Na_0.44_Mn_1−_ *_x_*Mg*_x_*O_2_ with *x* from 0 to 0.2. b) XRD patterns and Rietveld refinement of as‐prepared NMM‐0.05. c) XANES spectra of Mn K‐edge in NMM‐0.05 and standard manganese‐based metal oxide references (Mn_2_O_3_, LiMn_2_O_4_, and MnO_2_). d) Schematic illustration of NMM‐0.05 in the *a–b* plane with hinging site of Mn5 substituted by Mg^2+^. SEM images of NMM‐0.05 with scale bar of e) 10 and f) 1 µm.

Various ratios of Mg‐doped samples were successfully synthesized by solid‐state reaction (Na_0.44_MnO_2_, Na_0.44_Mn_0.95_Mg_0.05_O_2_, Na_0.44_Mn_0.9_Mg_0.1_O_2_, Na_0.44_Mn_0.85_Mg_0.15_O_2_, Na_0.44_Mn_0.8_Mg_0.2_O_2_, denoted as NMM‐0, NMM‐0.05, NMM‐0.1, NMM‐0.15, NMM‐0.2). The XRD patterns of synthesized samples with different doping ratios are shown in **Figure** **1**a and Figure S1, Supporting Information. NMM‐0 and NMM‐0.05 exhibit an orthorhombic tunnel structure with space group of *Pbam*. As the increase of Mg content over 0.05, the P2 phase begins to appear, mixed with the tunnel phase, as seen in NMM‐0.1. When the Mg content over 0.15, pure P2 phase can be observed in NMM‐0.15 and NMM‐0.2. The XRD refinements based on Rietveld method^[^ ^23^, ^24^, ^25^ ^]^ for NMM‐0.05 are shown in Figure 1b. The refined lattice constants are *a* = 9.082(3) Å, *b* = 26.432(5) Å, *c* = 2.824(8) Å. The increase of Mg content makes *b* expand while *a* and *c* slightly shrink, compared with those of NMM‐0 in Figure S2 and Table S1, S2, Supporting Information.

**Figure 2:**
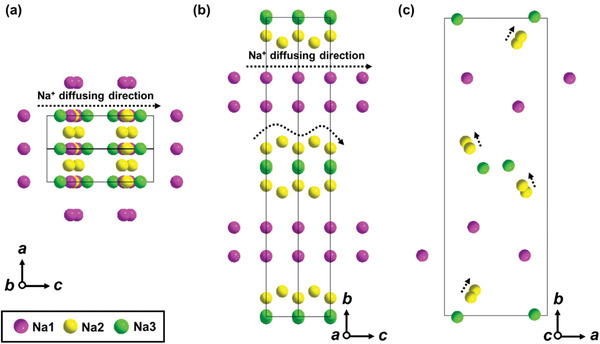
Sodium configuration in the tunnel structure and Na^+^ diffusing direction in different planes, a) *a*–*c* plane, b) *b*–*c* plane, c) *a*–*b* plane.

XAS was carried out to detect the valence state of Mn in NMM‐0.05 by comparing with relative Mn‐based metal oxide references (Mn_2_O_3_, LiMn_2_O_4_, and MnO_2_). As shown in Figure 1c, the average valance of Mn is about +3.6, which is consistent well with the expected stoichiometric ratio. Figure 1d shows the schematic illustration of the tunnel structure. The “Mn2‐Mn1‐Mn2‐Mn3‐Mn4‐Mn5” constructs the “S shape” channels, in which Na2 and Na1 are approximately half‐filled. While “Mn2‐Mn3‐Mn4‐Mn5‐Mn4‐Mn5” forms small channels with full‐filled Na1. The micro‐morphology of NMM‐0 and NMM‐0.05 obtained by scanning electron microscopy (SEM) are shown in Figure 1e,f; Figures S3 and S4, Supporting Information. It can be found that both NMM‐0 and NMM‐0.05 consist of rod‐shaped particles in the size range of 7–10 µm. Energy dispersive X‐ray spectroscopy (EDS) mappings indicate that Na, Mn, and Mg elements are distributed in the sample homogeneously. The atomic stoichiometry results are shown in Tables S3 and S4, Supporting Information, respectively, which agree well with the designed composition. To precisely confirm the actual ratios of metal ions in NMM‐0.05, ICP‐OES test was also conducted and the results are shown in Table S5, Supporting Information. The ratio of 0.441: 0.952: 0.048 for Na: Mn: Mg is very close to the expected stoichiometry. According to the results shown in Tables S1 and S2, Supporting Information, Mn1, Mn3, and Mn4 sites are occupied by Mn^4+^. While the Mn2‐O_6_ octahedral and Mn5‐O_5_ square pyramidal are occupied by Mn^3+^, which contributes to the charge center for Na^+^ insertion/extraction. According to the XRD refinement in Table S2, Supporting Information, Mg^2+^ is doped into the Mn5 sites, which is the only one Mn^3+^‐O_5_ square pyramidal. The Mn5‐O_5_ square pyramidal forms edge‐linked chains linking to two double and a triple octahedral chains, which leads to the formation of two types of tunnels. Thus, Mn5‐O_5_ plays as an important hinging role to tune lattice strains induced by insertion/extraction of Na ions. In NMM‐0.05, about 0.45 Mg ions substitute the Mn5 sites, and the formation of Mg 3s‐O 2p configuration introduces much flexible Mg‐O_5_ square pyramidal. The Mg_0.45_Mn_0.55_‐O_5_ square pyramidal enhances the hinge of small tunnel and “S shape” tunnel, suggesting the enlargement along *b* axis and the shrinkage of *a* and *c* parameters. In addition, this hinge can effectively tune the lattice strains and enhance the structure stability during repeated insertion/extraction of Na^+^. As for sodium ions shown in **Figure** **2**, Na1 occupied in the small tunnel. Sodium ions in Na2 and Na3 sites have a trigonal prismatic coordination with a Na—O bond in distance of 2.3–2.4 Å. During sodiation/desodiation process, Na ions transport sequentially along the *c* direction, following the arrow direction in Figure 2.

**Figure 3:**
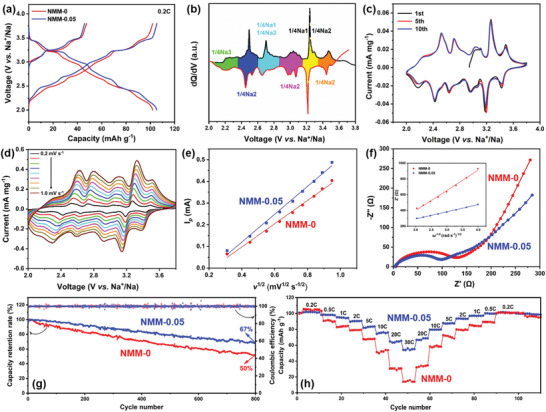
a) Charge/discharge curves of NMM‐0 and NMM‐0.05 between 2.0–3.8 V (versus Na^+^/Na) at 0.2 C (20 mA g^–1^). b) the differential capacity (d*Q*/d*V*) versus voltage profiles for the first discharge (black line) and second charge (red line). (Different filled colors present the amount of Na^+^ insertion/extraction). c) Cyclic voltammograms (CV) curves of NMM‐0.05 at a scan rate of 0.1 mV s^–1^. d) CV curves of NMM‐0.05 with various scanning rates from 0.2 to 1 mV s^–1^. e) Plot of the peak current (*I*_p_) as a function of the square root of scanning rates (*v* ^1/2^) calculated from CV in Figure 3d. f) EIS spectra of NMM‐0 and NMM‐0.05 after 80 cycles. Inset is the relationship between the real part of cell impedance (*Z*′) and the root of the angular frequency (*ω* ^–1/2^) in the low frequency region. g) Capacity retention rates of NMM‐0 and NMM‐0.05 after 800 cycles at 2 C. h) Rate capabilities of NMM‐0 and NMM‐0.05 from 0.2 to 30 C.

The electrochemical behaviors for NMM‐0.05 and NMM‐0 were evaluated by galvanostatic charge–discharge (GCD) and cyclic voltammogram (CV). **Figure** **3**a shows the GCD curves of NMM‐0 and NMM‐0.05 between 2.0–3.8 V at 0.2 C (20 mA g^–1^). In Figure 3a, NMM‐0 and NMM‐0.05 exhibit specific capacities of 46 and 47.2 mAh g^–1^ during the initial charge, respectively, extracted about half of the whole Na^+^ (0.22) from the tunnel host. In the following charge and discharge process, NMM‐0.05 delivers a reversible capacity of 105 mAh g^–1^, slightly higher than NMN‐0 (101 mAh g^–1^). Both the NMM‐0 and NMM‐0.05 electrodes exhibit similar six‐plateau characteristics, due to the complex phase evolution and sequential Na^+^ transferring for partial occupancy in tunnel host. To better understand the sequence of Na^+^ intercalation/deintercalation, the differential capacity versus voltage plot for NMM‐0.05 at 0.2 C in the first discharge (black) and second charge (red) is shown in Figure 3b. Six pairs of redox peaks suggest the sequential insertion/extraction of sodium ions, which are indexed as Na1, Na2, Na3 transferring from the small and “S shape” tunnel at different states of charge. The overall sequence of Na^+^ was quantitatively inserted or extracted due to the electrostatic repulsion among sodium ions.^[^ ^26^ ^]^ Figure 3c shows the typical CV curves of NMM‐0.05 in the first ten cycles, which is similar to the d*Q*/d*V* plot in Figure 3b. Six pairs of redox peaks in Figure 3c are quite symmetric and perfect overlapped for the first ten cycles, suggesting the effect of Mg doping on enhancing kinetics and lowering polarization. The enhanced kinetics and ionic diffusion of NMM‐0.05 were investigated by measuring the CV curves at different scanning rates. Figure S5, Supporting Information and Figure 3d are the CV curves of NMM‐0 and NMM‐0.05 at various scanning rates from 0.2 to 1.0 mV s^–1^. With the increase of scanning rates (*v*), the peak currents (*I*_p_) of the oxidation and reduction peaks also increase. Plotting *I*_p_ and the square root of scanning rate *v* ^1/2^ as a function in Figure 3e, a good linear character of *I*_p_ versus *v* ^1/2^ suggests the typical diffusion‐controlled behavior in sodiation/desodiation process.^[^ ^27^ ^]^ The diffusion coefficients of Na^+^ (*D*_Na+_) for NMM‐0 and NMM‐0.05 were calculated to be 2.897 × 10^–10^ and 3.496 × 10^–10^ cm^2^ s^–1^ based on Equation S1, Supporting Information, respectively. The improved *D*_Na+_ for NMM‐0.05 is attributed to the doping of Mg in tunnel structure. To further confirm the advantage of Mg doping for sodium storage kinetics, electrochemical impedance spectroscopy (EIS) were tested after 20 and 80 cycles at 0.2 C, as shown in Figure S6, Supporting Information and Figure 3f, Supporting Information. The *R*_ct_ value of NMM‐0.05, representing the resistance of charge transfer at the interfaces (semicircle in the middle frequency region), is significantly smaller than that of NMM‐0. According to Equations S2 and S3, Supporting Information, it is reconfirmed that *D*_Na+_ is effectively improved by the introduction of Mg, possibly due to the enlargement of *b* and the shrinkage in *a*–*c* plane. The calculated *D*_Na+_ values are listed in Table S6, Supporting Information. Meanwhile, GITT measurements were also performed to study the reaction kinetics of NMM‐0 and NMM0.05, shown in Figure S7, Supporting Information. It can be clearly seen that Mg‐doped NMM‐0.05 exhibits lower voltage polarization than NMM‐0, suggesting better kinetics of NMM‐0.05. In addition, NMM‐0.05 electrode exhibits superior long‐cycle stability, achieving 67% and 72% of the initial discharge capacity after 800 cycles at 2 and 5 C, respectively, while NMM‐0 decaying to only 50% and 47%, respectively, as shown in Figure 3g and Figure S8, Supporting Information. Figure S9, Supporting Information further illustrates that NMM‐0.05 can still keep excellent long‐cycle performance at even much higher current rate of 5, 10, and 20 C, reconfirming the effective strategy of Mg doping to improve the stability of the tunnel structure during long electrochemical cycling process. Figure 3h displays the rate performance of NMM‐0 and NMM‐0.05. With the increase of current density, the capacity attenuation of NMM‐0.05 is obviously improved than that of NMM‐0. Especially at 30 C, the NMM‐0.05 still maintains a specific capacity of 60, while only 20 mAh g^–1^ for undoped NMM‐0. After returning to 0.2 C, NMM‐0.05 can recover to 100 mAh g^–1^, almost same as the initial discharge capacity, demonstrating excellent rate capability. It is especially important that GCD curves of the NMM‐0.05 can exhibit stable multi‐platform characteristics as shown in Figure S10, Supporting Information, even the current density increases to 20 C, suggesting the excellent kinetics. The above results distinctly indicate that the doping of Mg has a significant improvement in electrochemical properties of the tunnel‐structured cathode material. The comprehensive electrochemical performance of NMM‐0.05 is outstanding compared with the reported tunnel‐structured cathode materials (Table S7, Supporting Information).

**Figure 4:**
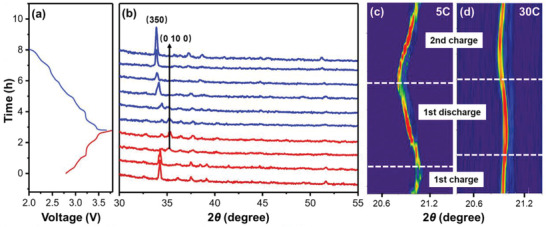
a) GCD curves of MMM‐0.05 electrode for taking ex situ XRD measurement at 0.2 C. b) XRD patterns of NMM‐0.05 at various charge/discharge states at 0.2 C (X‐ray wavelength: 1.5418 Å). Contour plots of the evolution in (350) peak during the initial cycling and the second charge process at c) 5 and d) 30 C, respectively (X‐ray wavelength: 0.6884 Å).

The structural evolution of NMM‐0.05 during the first charge and discharge is shown in **Figure** **4**. Figure 4a is the corresponding charge/discharge curves for capturing XRD data. As shown in Figure 4b, in the first charge process, all XRD peaks shift to higher two theta angles, suggesting the decrease of lattice parameters for NMM‐0.05. In addition, the intensity of (350) peak continues to decrease until 3.3 V, and then generates a new (0 10 0) peak, similar to the results of tunnel‐structured Na_0.44_MnO_2_ by Sauvage et al.^[^ ^28^ ^]^ During the discharge process, all the peaks move back to lower degree due to the intercalation of Na^+^ ions. The new formed (0 10 0) peak merges back into (350) peak, and disappeared completely after discharging below 2.7 V. At the same time, the intensity of (350) peak for NMM‐0.05 keeps increasing, suggesting the highly reversible structure evolution of NMM‐0.05 in the first cycle.

In order to monitor the structure evolution of NMM‐0.05 at high current rates, synchrotron‐based time‐resolved in situ XRD technique is carried out for NMM‐0.05 cycled at 5 and 30 C. The contour plots of the (350) peak during the initial cycle and the second charge process are presented in Figure 4c,d. At 5 C rate (Figure 4c), in situ XRD of NMM‐0.05 electrode exhibits similar peak evolution for (350) as that at 0.2 C (Figure 4b), implying high reversibility at 5 C rate. During 30 C cycling, the peak shift is less than that at 5 C rate, but the reversible shifting can still be observed. The highly reversible structure change behavior at high current rates is responsible for the superior rate capability of NMM‐0.05 shown in Figure 3h.

**Figure 5:**
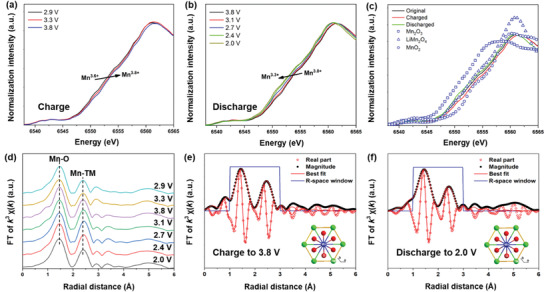
Mn K‐edge XANES of NMM‐0.05 at various states in the first a) charge and b) discharge. c) Mn K‐edge XANES of NMM‐0.05 at the pristine, fully charged (3.8 V), and fully discharged (2.0 V) states compared with Mn_2_O_3_ (Mn^3+^), LiMn_2_O_4_ (Mn^3.5+^), and MnO_2_ (Mn^4+^), respectively. d) Mn K‐edge Fourier transformed EXAFS spectrum of NMM‐0.05 at various states. Least‐square fits of the calculated FT‐EXAFS phase and amplitude functions to the experimental EXAFS spectra for the e) fully charged and f) fully discharged NMM‐0.05.

The charge compensation mechanism of NMM‐0.05 electrode during charge and discharge was investigated by synchrotron‐based XAS. The X‐ray absorption near edge structure (XANES), based on X‐ray photon‐induced electronic excitations from the core level to unoccupied electron states for Mn, were probed in **Figure** **5**a–c. When charging to 3.8 V, the energy of Mn K‐edge shifts to higher energy, indicating the oxidation of Mn^3.6+^ to higher valence state (about Mn^3.8+^), which agrees well with 0.22 Na^+^ extracted from NMM‐0.05 electrode in the first charge process. The desodiation process of Na_0.44_Mn_0.95_Mg_0.05_O_2_ to Na_0.22_Mn_0.95_Mg_0.05_O_2_ involves the oxidization of Mn2 and Mn5 from Mn^3+^ to Mn^4+^, requiring an electron removal from *e*_g_ orbit in the highest occupied molecular orbit (HOMO).^[^ ^26^ ^]^ In the discharge process (Figure 5b), the energy of Mn K‐edge continues to shift to lower energy. The Mn K‐edge energy of the sample discharged to 2.0 V is lower than that of the pristine NMM‐0.05, as compared in Figure 5c, because more Na^+^ were intercalated into the tunnel host. Compared with the standard references, it can be estimated that the Mn^3.8+^/Mn^3.3+^ redox couple contributes to the charge compensation, corresponding to the discharge capacity of 105 mAh g^–1^. In conclusion, for NMM‐0.05 electrode, Mn^3.6+^ is first oxidized to Mn^3.8+^ during the initial charge, then Mn^3.8+^/Mn^3.3+^ redox couple contributes to the charge compensation for the reversible electrochemical sodium storage.

The Fourier‐transformed extended X‐ray absorption fine structure (FT‐EXAFS) was used to analyze the evolution of bond lengths and coordination circumstances around Mn atoms. Mn K‐edge FT‐EXAFS spectra in various electrochemical states are shown in Figure 5d. The shift of the two peaks for Mn represent the first shell of Mn–O and the second shell of Mn‐TM. During the initial charge process, both Mn‐O and Mn‐TM distances decrease, consistence with the decrease of lattice parameter *a* and *b*. In the discharge process, both Mn‐O and Mn‐TM distances increase, and the values are even larger than those at the pristine state. Table S8, Supporting Information presents the quantitative structure parameters extracted from the FT‐EXAFS spectra in Figure 5e,f and Figure S11, Supporting Information. The bond length of the Mn‐O shrinks slightly during charge and then expands to be larger after discharging, illustrating the corresponding volume expansion and contraction in the *a–b* plane, which is consistent well with the XRD results.

**Figure 6:**
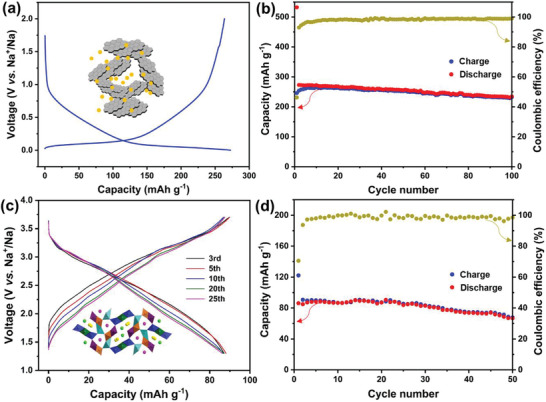
a) Galvanostatic discharge–charge curves and b) cycle performance of hard carbon at 0.2 C. c) Galvanostatic charge–discharge curves and d) cycle performance of NMM‐0.05//hard carbon full cell at 0.2 C.

The potential application of the NMM‐0.05 cathode was evaluated by assembling full cells with hard carbon as the anode. Hard carbon is considered as the most promising anode material for SIBs. It shows a slope at 1.0–0.1 V (versus Na^+^/Na) and a plateau at 0.1–0.01 V (versus Na^+^/Na) in the discharge process (**Figure** **6**a). The hard carbon anode we used shows excellent long‐term cycle performance with a reversible capacity of 250 mAh g^–1^ after 100 cycles, as shown in Figure 6b. Before constructing the full cells, the hard carbon was presodiated at 0.2 C, to eliminate the low initial coulombic efficiency. Figure 6c displays the GCD curves of the NMM‐0.05//hard carbon full cell. Different from multi‐plateau characteristics of NMM‐0.05//Na half‐cell in Figure 3a, the assembled full cell exhibits a smooth slope with an initial discharge capacity of 86.2 mAh g^–1^ (based on the mass of active material in cathode) and an average potential of 2.58 V. Figure 6d shows the cycle performance of the NMM‐0.05//hard carbon full cell. It exhibits the capacity retention of 77.5% after 50 cycles. The preliminary full‐cell performance proves that the NMM‐0.05 cathode is feasible and promising for potential application.

**Figure 7:**
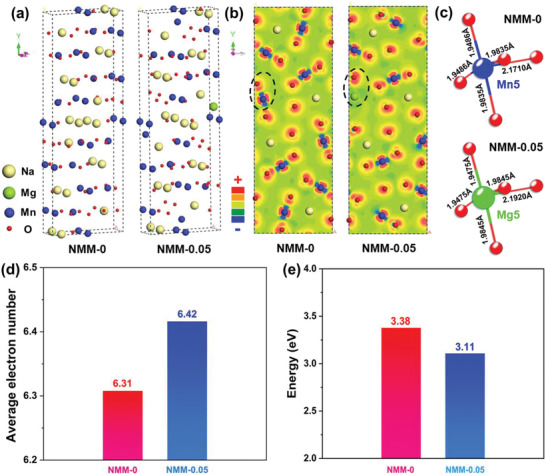
a) Optimized structure model of NMM‐0 and NMM‐0.05. b) Differential charge analysis of NMM‐0 and NMM‐0.05 in *x–y* plane (Red “+” represents electron gaining trend, while blue “‐” means electron loss tendency). c) Bond length evolution of Mn5‐O in NMM‐0 and Mg5‐O in NMM‐0.05, deriving from the XRD refinement results in pristine NMM‐0 and NMM‐0.05. d) Atom population analysis of the average electron number of Mn in NMM‐0 and NMM‐0.05. e) Desodiation energy comparison of NMM‐0 and NMM‐0.05.

To further elucidate the advantage of Mg doping in tunnel‐Na_0.44_MnO_2_, DFT calculations were performed to study the average electron number of Mn near the Fermi level and desodiation energy in NMM‐0 and NMM‐0.05. **Figure** **7**a shows the optimized structure model used for DFT calculation. It can be clearly seen that the basic tunnel structure remains unchanged after Mg doping. Differential charge analysis from *x–y* plane was conducted to explore the trend of electron gain and loss in NMM‐0 and NMM‐0.05, as shown in Figure 7b. Red color suggests the oxygen atoms are easier to gain electrons. On the contrary, blue color along “O‐Mn‐O” direction represents that Mn atoms tend to lose electrons. It is worth to note that the electron density of oxygen atoms along “O‐Mg‐O” direction (cycled region) increases after Mg doping, suggesting that Mg doping can elevate electron‐captured ability of these oxygen atoms. Figure 7c compares the bond length of Mn5‐O in NMM‐0 and Mg5‐O in NMM‐0.05. In Mn5(Mg5)‐O5 pyramid, one Mn(Mg)—O bond length increases from 2.1710 to 2.1920 Å, other two Mn(Mg)—O bond lengths increase slightly from 1.8935 to 1.8945 Å, while the last two bond lengths decrease from 1.9486 to 1.9475 Å. The distortion of Mn/Mg‐O_5_ pyramid can generate a stable and flexible Mn5(Mg)‐O_5_ hinge after Mg doping, which can relieve the lattice strain preferably and be well adapted to the structure change during Na^+^ deintercalation/intercalation. In addition, with the assistance of electron population analysis in Figure 7d; Tables S9 and S10, Supporting Information, it can be concluded that the average electron number of Mn near the Fermi level in NMM‐0.05 is increased from 6.31 to 6.42 after Mg^2+^ doping. Therefore, Mg^2+^ doping can lift more Mn 3*d* electrons closer to the Fermi level, facilitating greater Mn participation in the redox reaction and promoting electron transferring over the range of Na content,^[^ ^29^ ^]^ leading to superior rate performance of NMM‐0.05. In addition, the calculated desodiation energy shown in Figure 7e decreased from 3.38 eV for NMM‐0 to 3.11 eV for NMM‐0.05, suggesting that the energy barrier for Na^+^ deintercalation is obviously relieved by Mg^2+^ doping in the tunnel structure. The DFT calculations prove the enhancement of thermodynamics and kinetics properties for sodium storage in NMM‐0.05, compared with NMM‐0.

The above results demonstrate that Mg^2+^ doping in Na_0.44_MnO_2_ is an effective strategy to improve electrochemical performance and kinetics for tunnel‐structured cathode materials. In previous studies, Mg^2+^ has been widely used as the dopant in layered cathode materials. Compared with Li trigging mechanism in Li‐rich materials, Mg doping in layered cathode materials can also induce anionic redox, suppress oxygen loss.^[^ ^19^, ^20^, ^21^ ^]^ In this work, about 0.45 Mg^2+^ substitute the Mn5 sites to form tunnel‐structured Na_0.44_Mn_0.95_Mg_0.05_O_2_ as shown in Figure 1d. The Mg_0.45_Mn_0.55_‐O_5_ square pyramidal forms edge‐linked chains (two double and a triple octahedral chains), bridging the small tunnel and “S shape” tunnel together. Thus, the Mg_0.45_Mn_0.55_‐O_5_ square pyramidal plays an important hinging role to relieve lattice strains induced by Na^+^ insertion/extraction. The doping of Mg^2+^ can effectively decrease the desodiation energy from 3.38 eV (for NMM‐0) to 3.11 eV (for NMM‐0.05). As a result, NMM‐0.05 exhibits superior long‐cycle stability, retaining 67% of the initial discharge capacity after 800 cycles at 2 C, comparing only 50% for NMM‐0. Especially, NMM‐0.05 electrode exhibits promising fast charge/discharge behaviors, keeping 70% of the initial discharge capacity after 800 cycles at 20 C. According to CV and EIS results, Mg^2+^ doping in the tunnel structure can facilitate sodium‐ion diffusion. Differential charge calculation and atom population analysis of the average electron number of Mn also show that NMM‐0.05 is more electron‐abundant in Mn 3*d* orbits near the Fermi level, which leads to the excellent rate capability of NMM‐0.05. During the first charge process, 1/4 Na2 and 1/4 Na3 deintercalated at 2.9–3.8 V mainly due to the oxidation of Mn2 and Mn5, involving the redox reaction from Mn^3+^ to Mn^4+^. Mg^2+^ doping can also lower the active energy for electron transmission from *e*_g_ orbit of Mn2 and Mn5 in the HOMO.

The rate‐dependent structure evolution for tunnel‐type cathode material during charging/discharging was revealed by using time‐resolved in situ XRD for the first time. In LIBs, intermediate phases have been observed in LiMn_0.33_Ni_0.33_Co_0.33_O_2_ between H1 and H2 phases at high rate charging process.^[^ ^30^ ^]^ In SIBs system, P2‐layered Na_0.7_Mg_0.05_[Mn_0.6_Ni_0.2_Mg_0.15_]O_2_ also exhibits multiple intermediate phases due to the sluggish kinetics during sodiation/desodiation.^[^ ^19^ ^]^ However, in tunnel‐structured cathode, the special “S shape” channels are easier for Na^+^ transportation, without new intermediate phase occurring at high current density, as shown in Figure 4c,d. Although Na^+^ can be intercalated into and deintercalated from tunnel structure, the extra driving force provided by high current rate should be beneficial for overcoming the energy barrier of phase transition. The particular sequence of Na^+^ diffusion may also play a very important role to suppress the inhomogeneity of sodium‐ion distribution in the tunnel lattice, avoiding forming the intermediate phases. The highly reversible structure evolution at high current can be responsible for the excellent rate capability of NMM‐0.05.

## 3 Conclusion

In summary, it is revealed that Mg^2+^ doping in the Mn5 site of tunnel‐structured Na_0.44_MnO_2_ cathode can not only facilitate Na^+^ transportation, but also decrease the sodium deintercalation energy and increase electrons near the Fermi level, thus improving cycle stability and rate capability effectively. Synchrotron‐based time‐resolved in situ XRD confirms that NMM‐0.05 undergoes single phase evolution during fast charge and discharge processes without the existence of intermediate phases, which is quite different from those observed in layer‐structured cathode materials. As a result, NMM‐0.05 cathode exhibits superior long‐cycle stability and rate capability. This work not only provides a promising strategy for improving electrochemical performance of tunnel‐structured cathode materials, but also gives a hint that the fast‐charging ability of cathode materials could be improved by constructing Li or Na ordering sequence in crystal channels, which can effectively restrict the inhomogeneity of Li^+^ and Na^+^ during ion transportation and suppress the irreversible phase transition.

## 4 Experimental Section

##### Material Synthesis

A series of Mg^2+^ doped Na_0.44_Mn_1−_ *_x_*Mg*_x_*O_2_ (Na_0.44_MnO_2_, Na_0.44_Mn_0.98_Mg_0.02_O_2_, Na_0.44_Mn_0.95_Mg_0.05_O_2_, Na_0.44_Mn_0.925_Mg_0.075_O_2_, Na_0.44_Mn_0.9_Mg_0.1_O_2_, Na_0.44_Mn_0.85_Mg_0.15_O_2_, Na_0.44_Mn_0.8_Mg_0.2_O_2_, denoted as NMM‐0, NMM‐0.02, NMM‐0.05, NMM‐0.075, NMM‐0.1, NMM‐0.15, NMM‐0.2) were synthesized by traditional solid‐reaction method. Stoichiometric Na_2_CO_3_ (5 wt% excess to compensate sodium evaporation), Mn_2_O_3_, MgO were ball milled in ethanol for half an hour. Then the homogenous mixture was heated at 850 °C for 20 h with a heating rate of 5 °C min^–1^ in oxygen atmosphere.

##### Material Characterization

The crystal structure of the synthesized samples was determined by XRD (Bruker D8 Advance, Germany) with Cu‐K*α* radiation (*λ* = 0.154 nm) at 40 kV, 40 mA. Morphologies of the materials were obtained by field emission scanning electron microscopy (Cambridge S‐360). XAS was performed at beamline BL14W1 of Shanghai Synchrotron Radiation Facility and beamline 7‐BM (QAS) of National Synchrotron Light Source II. Mn K‐edge XAS spectra were collected in transmission mode. The XAS data was processed using Athena and Artemis software packages. In situ XRD was carried out at beamline BL14B1 (λ = 0.6884 Å) of SSRF.

##### Electrochemical Performance

Electrodes were prepared by fully blending the active cathode material (70 wt%), polyvinylidene fluoride (10 wt%) and carbon black (20 wt%) in *N*‐methyl‐2‐pyrrolidone as a dispersing agent. The slurry was cast on aluminum foils and dried in a vacuum oven at 70 °C for 12 h. Electrochemical tests were carried out in coin cell (CR‐2032). The electrolyte solution consists of 1 m NaClO_4_ with addition of ethylene carbonate, propylene carbonate and 5 wt% fluoroethylene carbonate. Sodium metal was used as the counter electrode. All the half‐cells were cycled within the voltage range of 2.0–3.8 V versus Na^+^/Na at 25 °C on a Land CT2001A battery test system (Wuhan, China). EIS was evaluated with a frequency range from 10 mHz to 1 MHz. CV tests were performed at the scanning rate of 0.2–1.0 mV s^–1^ between 2.0–3.8 V versus Na^+^/Na. The full cell was assembled with NMM‐0.05 as the cathode and hard carbon as the anode. To eliminate the relative low Coulombic efficiency of hard carbon and NMM‐0.05 in the initial cycle, both cathode and anode were precycled. In order to balance the capacity between cathode and anode in a full cell, the N:P ratio was set to ≈1.2. The mass loading of cathode and anode materials were 2.77 and 1.33 mg cm^–2^, respectively.

##### Theoretical Calculations

DFT calculations were employed to study the Na deintercalation energy, differential charge analysis and atom population analysis of the two structures (NMM‐0 and NMM‐0.05). The theoretical calculations were conducted in Cambridge Sequential Total Energy Package (CASTEP) of Materials Studio (Accelrys Inc.). The structures were built according to the XRD refinement results and a 1 × 1 × 1 supercell for each structure was built separately. The desodiation energy was defined as: *E* = (*E*_NMM‐0/0.05_ + *nE*_Na_ − *E*_NMM‐0/0.05*_)/*n*, (*E*_NMM‐0/0.05_ means the energy after desodiation; *E*_NMM‐0/0.05*_ means the energy before desodiation; *E*_Na_ means the energy of Na and *n* is the number of Na that deintercalated). Geometry optimizations of the above structures were conducted separately. The calculation quality was set to fine and the GGA‐PBE functional was employed. The convergence tolerance was set as: 1 × 10^–5^ eV atom^–1^ for energy, 0.03 eV Å^–1^ for max force, 0.05 GPa for max stress, 0.001 Å for max displacement. The cut‐off energy was 598.7 eV and ultra‐soft pseudopotential was employed. The *k*‐point was set to 2 × 1 × 5. Koelling–Hamon relativistic treatment was chosen to deal with heavy atoms. Self‐consistent field tolerance was set to 1 × 10^–6^ eV atom^–1^.

## Conflict of Interest

The authors declare no conflict of interest.

## Supporting information

Supporting Information

## Data Availability

Research data are not shared.
