# Supplementary material for: Boosting Reversibility of Mn‐Based Tunnel‐Structured Cathode Materials for Sodium‐Ion Batteries by Magnesium Substitution
Source: Adv Sci (Weinh). 2021 Feb 18;8(9):2004448. doi: 10.1002/advs.202004448 (PMC8097362; doi:10.1002/advs.202004448)
Supplement: Supplementary file 1 — Supporting Information [file ADVS-8-2004448-s001.pdf]

© 2021 Wiley-VCH GmbH

ADVANCED SCIENCE Open Access

Supporting Information

for *Adv. Sci.,* DOI: 10.1002/advs.202004448

Boosting reversibility of Mn-Based tunnel-structured cathode materials for sodium-ion batteries by magnesium substitution

Xun-Lu Li, Jian Bao, Yi-Fan Li, Dong Chen, Cui Ma, Qi-Qi Qiu, Xin-Yang Yue, Qin-Chao Wang and Yong-Ning Zhou**

Supporting Information

Boosting reversibility of Mn-based tunnel-structured cathode materials for sodium-ion batteries by magnesium substitution

Xun-Lu Li, Jian Bao, Yi-Fan Li, Dong Chen, Cui Ma, Qi-Qi Qiu, Xin-Yang Yue, Qin-Chao Wang and Yong-Ning Zhou**

$$I_p = 2.69\times10^5 n^{3/2} A D^{1/2} v^{1/2} C_0 \qquad \text{Equation S1}$$

Note: I_p is the peak currents of the oxidation and reduction peaks in the CV, D is the diffusion coefficient of Na^+. n is the number of electrons per reaction species, A is the area of the electrode, C_0 is the concentration of Na^+ in the lattice, v is scan rates.

$$D_{Na+} = R^2T^2/2A^2n^4F^4C^2\sigma^2 \qquad \text{Equation S2}$$

Note: R is the gas constant (8.314 J K^{-1} mol^{-1}), T is the test temperature (298 K), A is the surface area of the electrode. n is the number of the electrons per molecule during oxidization, F is the Faraday constant (96485 C mol^{-1}), C is molar concentration of Na^+, σ is the Warburg factor (Ω $s^{-1/2}$) associated with Z' (real part of the cell impedance, Ω) and ω (the angular frequency, rad s^{-1})

$$Z' = R_e + R_{ct} + \sigma\omega^{-1/2} \qquad \text{Equation S3}$$

Note: R_e is the resistance of the electrolyte, R_{ct} is the charge transfer resistance[S1].

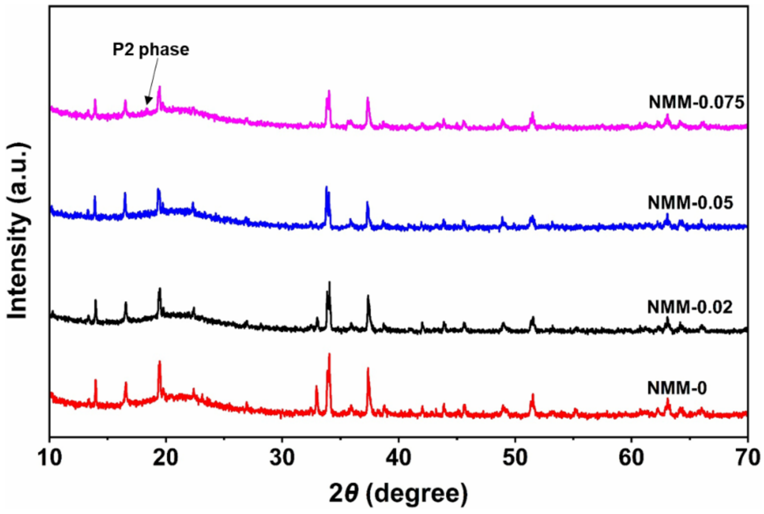

Figure S1. XRD patterns of $Na_{0.44}MnO_2$ (NMM-0), $Na_{0.44}Mn_{0.98}Mg_{0.02}O_2$ (NMM-0.02), $Na_{0.44}Mn_{0.95}Mg_{0.05}O_2$ (NMM-0.05), and $Na_{0.44}Mn_{0.925}Mg_{0.075}O_2$ (NMM-0.075).

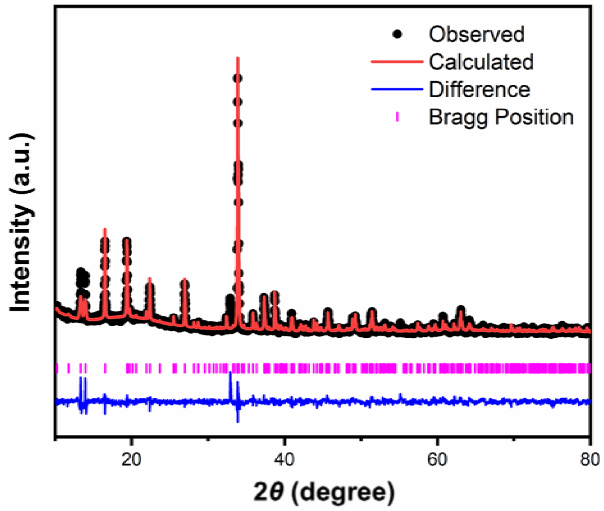

Figure S2. Rietveld refinement of the XRD pattern for $Na_{0.44}MnO_2$.

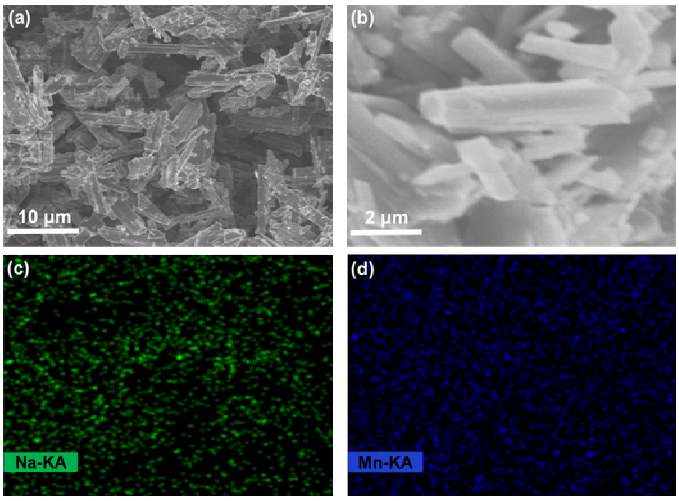

Figure S3. SEM images of a, b) NMM-0 and corresponding EDX mapping of c) Na and d) Mn.

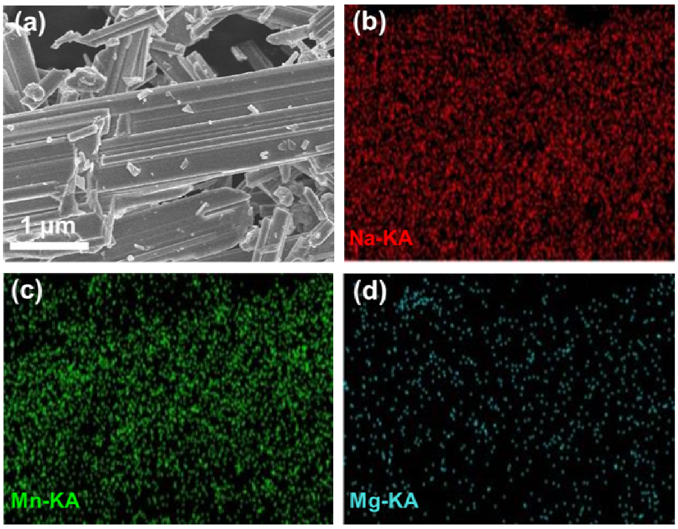

Figure S4. SEM image of a) NMM-0.05 and corresponding EDX mapping of b) Na, c) Mn and d) Mg.

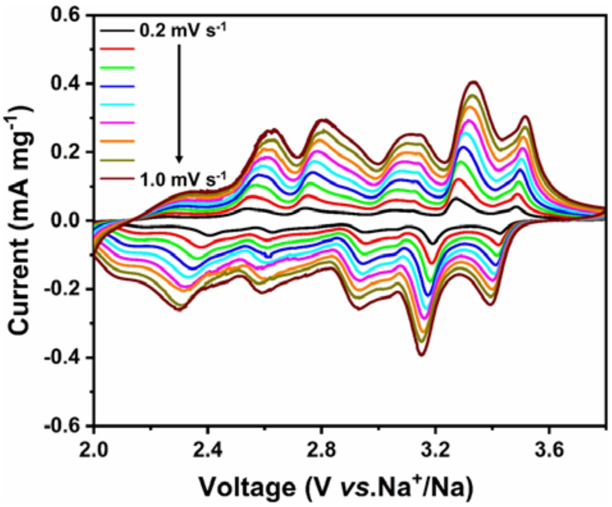

Figure S5. CV curves of NMM-0 with scan rates increasing from 0.2 to 1 mV s^{-1}.

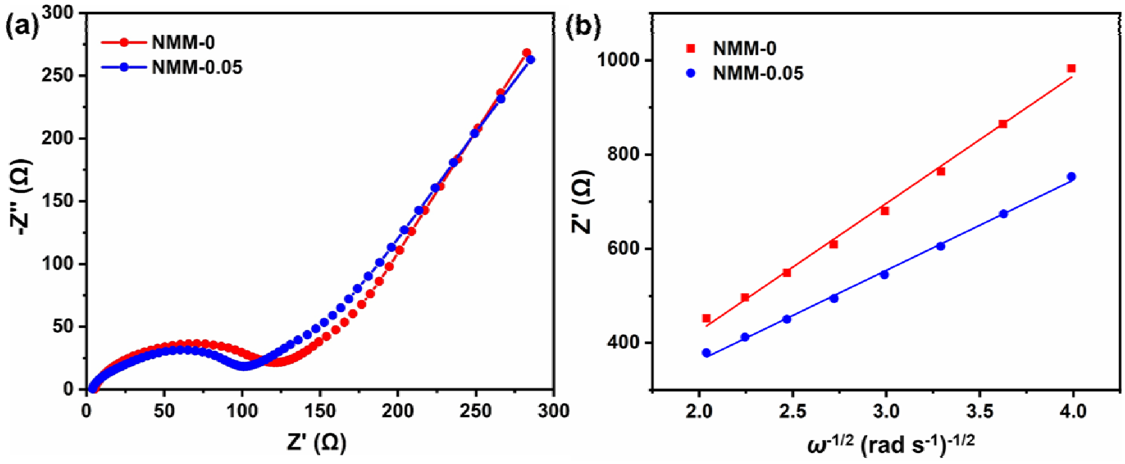

Figure S6. a) EIS spectra of NMM-0 (red) and NMM-0.05 (blue) after 20 cycles. b) Plots of Z' as a function of $\omega^{-1/2}$ in the low frequency region.

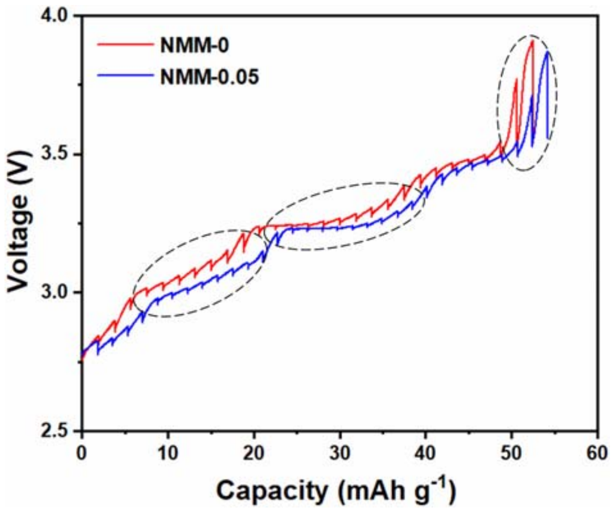

Figure S7. GITT curves of NMM-0 and NMM-0.05 in the first charge process.

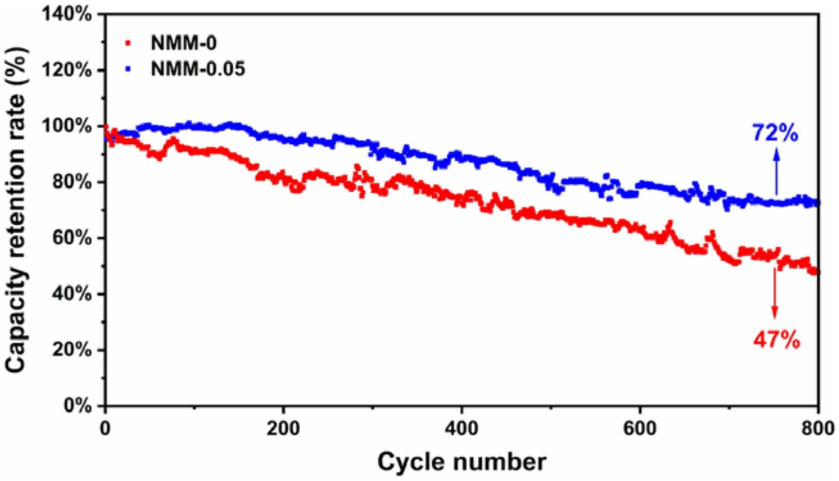

Figure S8. The capacity retention rates of NMM-0 and NMM-0.05 at 5C.

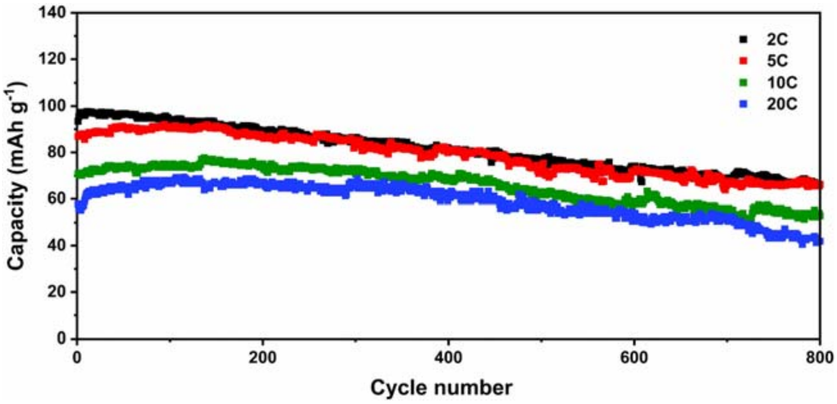

Figure S9. Long cycle performance of NMM-0.05 at 2C (black), 5C (red), 10C (green), and 20C (blue).

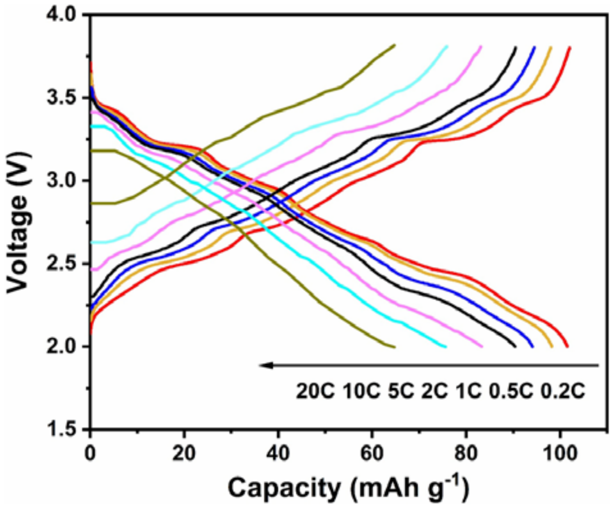

Figure S10. The galvanostatic GCD curves of NMM-0.05 between 2.0-3.8 V *vs.* Na^+/Na at different current densities.

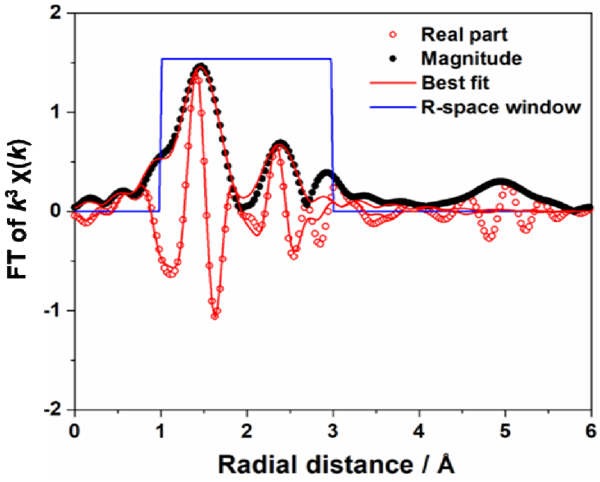

Figure S11. Least-square fits of the calculated FT-EXAFS phase and amplitude functions to the experimental EXAFS spectra for the pristine NMM-0.05.

Table S1. Crystallographic parameters of $Na_{0.44}MnO_2$ refined by Rietveld method.

Space group: *Pbam* (No. 55)						
**Atom**	**element**	**Mult.**	***x***	***y***	***z***	**Occupancy**
Mn1	Mn^{4+}	2	0	0.5	0	1
Mn2	Mn^{3+}	4	0.3570	0.0934	0.5	1
Mn3	Mn^{4+}	4	0.0350	0.1005	0	1
Mn4	Mn^{4+}	4	0.3490	0.3067	0.5	1
Mn5	Mn^{3+}	4	0.0260	0.3080	0	1
Na6	Na^{+}	4	0.2010	0.1890	0	1
Na7	Na^{+}	4	0.2960	0.4090	0.5	1
Na8	Na^{+}	4	0.1240	0.0038	0	1
O9	O^{2-}	4	0.3540	0.0020	0.5	1
O10	O^{2-}	4	0.2600	0.0930	0	1
O11	O^{2-}	4	0.0660	0.1920	0.5	1
O12	O^{2-}	4	0.5290	0.1830	0.5	1
O13	O^{2-}	4	0.1790	0.3020	0.5	1
O14	O^{2-}	4	0.3920	0.3017	0	1
O15	O^{2-}	4	0.2300	0.4000	0	1
O16	O^{2-}	4	0.5140	0.0870	0	1
O17	O^{2-}	4	0.4850	0.4100	0.5	1
a=9.100(6)	b=26.394(5)	c=2.828(7)	V=679.4(9)	R_p=1.5%	R_{wp}=2.53%	

Table S2. Crystallographic parameters of $Na_{0.44}Mn_{0.95}Mg_{0.05}O_2$ refined by Rietveld method.

Space group: *Pbam* (No. 55)

**Atom**	**element**	**Mult.**	***x***	***y***	***z***	**Occupancy**
Mn1	Mn^{4+}	2	0	0.5000	0	1
Mn2	Mn^{3+}	4	0.5130	0.0533	0.5	1
Mn3	Mn^{4+}	4	0.0297	0.1006	0	1
Mn4	Mn^{4+}	4	0.3570	0.3050	0.5	1
Mg5	Mg^{2+}	4	0.0380	0.2956	0	0.45
Mn5	Mn^{3+}	4	0.0380	0.2956	0	0.55
Na6	Na^{+}	4	0.2460	0.2058	0	1
Na7	Na^{+}	4	0.1980	0.3878	0.5	1
Na8	Na^{+}	4	0.1360	0.0200	0	1
O9	O^{2-}	4	0.1790	0.0030	0.5	1
O10	O^{2-}	4	0.2550	0.0912	0	1
O11	O^{2-}	4	0.1240	0.1928	0.5	1
O12	O^{2-}	4	0.3880	0.2173	0.5	1
O13	O^{2-}	4	0.0950	0.2925	0.5	1
O14	O^{2-}	4	0.3090	0.2940	0	1
O15	O^{2-}	4	1.5270	0.7028	0	1
O16	O^{2-}	4	0.3510	0.1290	0	1
O17	O^{2-}	4	0.4160	0.4134	0.5	1

a=9.082(3) b=26.432(5) c=2.824(8) V=678.16(4) R_p=3.48% R_{wp}=4.60%

Table S3. EDX results of NMM-0.

**Element**	**Series**	**Atom ratio [at%]**	**Deviation [%]**
Na	K	31.48	0.92
Mn	K	68.52	0.92

Table S4. EDX results of NMM-0.05.

Element	Series	Atom ratio [at%]	Deviation [%]
Na	K	32.67	2.0
Mn	K	63.52	1.5
Mg	K	3.81	0.4

Table S5. ICP results of the synthesized $Na_{0.44}Mn_{0.95}Mg_{0.05}O_2$.

Na	Mn	Mg
0.441	0.952	0.048

Table S6. D_{Na+} values of NMM-0 and NMM-0.05 at the 20th cycle and 80th cycle, respectively.

Sample	20th D_{Na+} (cm^2 s^{-1})	80th D_{Na+} (cm^2 s^{-1})
NMM-0	7.314×10^{-11}	8.660×10^{-11}
NMM-0.05	1.386×10^{-10}	5.468×10^{-10}

Table S7 Electrochemical performance comparison of NMM-0.05 with other tunned-structured cathode materials for sodium-ion batteries.

**Samples**	**Specific capacity (mAh g^{-1}@C)**	**Rate capability (mAh g^{-1}@C)**	**Cycling stability (%@cycle No./C)**	**Voltage range (V)**	**Ref.**
$Na_{0.61}[Mn_{0.27}Fe_{0.34}Ti_{0.39}]O_2$	98@0.1	43@1	90@100/0.2	2.6~4.2	[S2]
$Na_{0.66}[Mn_{0.66}Ti_{0.34}]O_2$	74@0.1	-	86@400/0.1	2.5~3.9	[S3]
$Na_{0.44}MnO_2$ nanofiber	118@0.42	69.5@10	67@140/0.42	1.5~4.0	[S4]
$Na_{0.5}MnO_2$	115@0.1	100@2	95@30/0.33	2.0~4.0	[S5]
$Na_{0.66}[Mn_{0.66}Ti_{0.34}]O_{1.94}F_{0.06}$	97@0.2	65@20	87@1000/2	1.5~3.8	[S6]
**NMM-0.05**	105@0.2	60@30	72@800/5	2.0~3.8	**This work**

Table S8. Evolution of Mn K-edge EXAFS structural parameters at various states for NMM-0.05.

Samples	Path	r / Å	σ^2 / 10^{-3} Å^2	ΔE / eV	R
Pristine	Mn-O	1.892 ± 0.076	1.232 ± 1.694	-1.922 ± 2.577	0.001
	Mn-TM	2.786 ± 0.099	9.202 ± 1.857	-14.654 ± 3.564	
Fully charged	Mn-O	1.874 ± 0.075	0.336 ± 0.800	-0.075 ± 1.042	0.001
	Mn-TM	2.762 ± 0.059	5.838 ± 0.722	-7.770 ± 1.498	
Fully discharged	Mn-O	1.902 ± 0.065	1.435 ± 2.375	-0.367 ± 4.144	0.001
	Mn-TM	2.821 ± 0.124	10.402 ± 3.476	-18.602 ± 8.730	

r: bond length; σ^2: Debye-Waller factor (disorder); ΔE: inner shell potential shift; R: R-factor

Table S9. Atom population analysis of NMM-0.

**Atom**	**Number**	***s***	***p***	***d***	**Total**
Mn	1	0.58	0.52	5.23	6.33
Mn	2	0.58	0.52	5.23	6.33
Mn	3	0.59	0.46	5.2	6.25
Mn	4	0.53	0.51	5.24	6.28
Mn	5	0.59	0.46	5.2	6.25
Mn	6	0.53	0.51	5.24	6.28
Mn	7	0.59	0.46	5.2	6.25
Mn	8	0.53	0.51	5.24	6.28
Mn	9	0.59	0.46	5.2	6.25
Mn	10	0.53	0.51	5.24	6.28
Mn	11	0.59	0.53	5.31	6.44
Mn	12	0.61	0.46	5.19	6.25
Mn	13	0.59	0.53	5.31	6.44
Mn	14	0.61	0.46	5.19	6.25
Mn	15	0.59	0.53	5.31	6.44
Mn	16	0.61	0.46	5.19	6.25
Mn	17	0.59	0.53	5.31	6.44
Mn	18	0.61	0.46	5.19	6.25

Table S10. Atom population analysis of NMM-0.05.

Atom	Number	*s*	*p*	*d*	Total
Mn	1	0.63	0.28	5.5	6.42
Mn	2	0.63	0.28	5.5	6.53
Mn	3	0.63	0.28	5.5	6.41
Mn	4	0.63	0.28	5.5	6.51
Mn	5	0.63	0.28	5.5	6.37
Mn	6	0.63	0.28	5.5	6.51
Mn	7	0.55	0.36	5.37	6.29
Mn	8	0.58	0.38	5.37	6.32
Mn	9	0.62	0.23	5.54	6.39
Mn	10	0.57	0.37	5.4	6.35
Mn	11	0.58	0.45	5.42	6.45
Mn	12	0.57	0.36	5.4	6.33
Mn	13	0.58	0.41	5.38	6.36
Mn	14	0.62	0.38	5.37	6.37
Mn	15	0.61	0.48	5.47	6.55
Mn	16	0.58	0.38	5.43	6.4
Mn	17	0.59	0.48	5.45	6.51

References

[S1] J. G. Duan, C. Wu, Y. B. Cao, K. Du, Z. D. Peng, G. R. Hu, *Electrochim. Acta* **2016**, *221*, 14.

[S2] S. Y. Xu, Y. S. Wang, L. B. Ben, Y. C. Lyu, N. N. Song, Z. Z. Yang, Y. M. Li, L. Q. Mu, H. T. Yang, L. Gu, Y. S. Hu, H. Li, Z. H. Cheng, L. Q. Chen, X. J. Huang, *Adv. Energy Mater* **2015**, *5*, 1501156.

[S3] Y. S. Wang, L. Q. Mu, J. Liu, Z. Z. Yang, X. Q. Yu, L. Gu, Y. S. Hu, H. Li, X. Q. Yang, L. Q. Chen, X. J. Huang, *Adv. Energy Mater* **2015**, *5*, 1501005.

[S4] B. Fu, X. Zhou, Y. P. Wang, *J. Power Sources* **2016**, *310*, 102.

[S5] K. Y. Shen, M. Lengyel, L. Wang, R. L. Axelbaum, *MRS Commun* **2017**, *7*, 74.

[S6] Q. C. Wang, Q. Q. Qiu, N. Xiao, Z. W. Fu, X. J. Wu, X. Q. Yang, Y. N. Zhou, *Energy Storage Mater* **2018**, *15*, 1.
